# Endophytic Fungi Isolated from Plants Growing in Central Andean Precordillera of Chile with Antifungal Activity against *Botrytis cinerea*

**DOI:** 10.3390/jof6030149

**Published:** 2020-08-26

**Authors:** Araceli Vidal, Rodolfo Parada, Leonora Mendoza, Milena Cotoras

**Affiliations:** Faculty of Chemistry and Biology, University of Santiago of Chile, Bernardo O’Higgins Avenue 3363, Estación Central, Santiago 9160000, Chile; araceli.vidal@usach.cl (A.V.); rodolfo.paradaf@usach.cl (R.P.)

**Keywords:** *Botrytis cinerea*, endophytic fungi, antifungal activity

## Abstract

*Botrytis cinerea* is an important phytopathogenic fungus affecting the fruit production around the world. This fungus is controlled mainly by using synthetic fungicides, but many resistant isolates have been selected by the indiscriminate use of fungicides. Endophytic fungi or secondary metabolites obtained from them become an alternative method of control for this fungus. The aim of this work was to identify endophytic fungi with antifungal activity against the plant pathogenic fungus *B. cinerea* isolated from plants from Central Andean Precordillera of Chile. Three endophytic fungi (Ac1, Lc1 and Ec1) with antifungal activity against *B. cinerea* were isolated from native and endemic plants growing in Central Andean Precordillera of Chile. The isolates Lc1 (isolated from *Lithraea caustica*) and Ac1 (isolated from *Acacia caven*) were identified as *Alternaria* spp. and the isolate Ec1 (isolated from *Echinopsis chiloensis*) was identified as *Aureobasidium* spp. The isolated endophytic fungi would inhibit *B. cinerea* through the secretion of diffusible and volatile compounds affecting the mycelial growth, conidia germination and interestingly, it was also shown that the volatile compounds produced by the three isolated endophytic fungi suppressed the sporulation of *B. cinerea*.

## 1. Introduction

*Botrytis cinerea* is a filamentous necrotrophic fungus that infects more than 250 plant species around the world, and it is the causal agent of “gray mould” disease [1]. Gray mold causes great economic losses in fruit production due to decay of crops and it produces biochemical changes that alter the organoleptic quality of fruits [1]. The estimated lost ranging from USD 10 billion to USD 100 billion [1]. Traditional control of this disease is realized through cultural management, elimination of remains of infected plants, and by the use of synthetic fungicides and biofungicides, including biological control [2]. Currently, the management of the disease is affected by the selection of *B. cinerea* isolates resistant to fungicides [3]. In a study realized in table grapes of Thompson Seedless variety in the Central Valley of Chile, it was demonstrated that only 1% of the *B. cinerea* isolates was sensitive to all of fungicide families [4]. Therefore, new alternatives for the control of this disease are necessary. An alternative is the use of endophytic fungi or secondary metabolites obtained from them [5]. Endophytic fungi are organisms that can live inside plants without causing symptoms of disease in their host. These fungi can have a great impact on host plants, because they can increase the plant tolerance to biotic and abiotic stress [6]. Plant tolerance to biotic stress can be increased because some endophytic fungi produce compounds with antimicrobial activity [5]. Numerous endophytic fungi, among them, species of the genera *Aspergillus*, *Aureobasidium*, *Alternaria*, *Phoma*, *Fusarium*, *Trichoderma*, *Penicillium*, that significantly suppress the growth of *B. cinerea* have been isolated from different plant species [7]. There are different criteria for selection of plants as a source of isolation of fungal endophytes with a considerable inhibition effect to *B. cinerea* growth; one of them is to look for endemic plants, because these plant species are distributed in an exclusive reduced geographical location; therefore, it is expected that these plants possess specific endophytes [8]. Central Andean Precordillera of Chile has a Mediterranean climate called “Chilean scrub”. This climate is characterized by rainy winters and long dry summers. Mediterranean climate is only present in five regions of the world [9,10]. Therefore, plants growing in these regions encounter in conditions of abiotic stress [11].

The aim of this work was to identify endophytic fungi obtained from different tissue types of various plant families from Central Andean Precordillera of Chile producing volatile or diffusible compounds with antifungal activity against the plant pathogenic fungus *B. cinerea.*

## 2. Materials and Methods

### 2.1. Collection of Plant Material

Samples of different tissue types (stem, leaves or flowers) from healthy plants growing in El Ingenio, Cajón del Maipo in central Andean Precordillera of Chile (Latitude: −33°46′8.76″; Longitude: −70°16′35.03″ and Altitude 1200 m) was collected in August 2017. The study material was obtained from endemic and native species of different plant families (*Calceolaria polifolia* (Cp), *Lithraea caustica* (Lc), *Colliguaja odorifera* (Co), *Echinopsis chiloensis* (Ec), *Tropaeolum tricolor* (Tt), *Puya chilensis* (Pc) and *Acacia caven* (Ac)). Stems, leaves, or flowers were excised from one plant of each plant species with a sterile scalpel and placed in sterile Falcon tubes at 4 °C until use. In the case of *E. chiloensis,* stem pieces of 5 cm were collected for endophyte isolation.

### 2.2. Isolation of Endophytic Fungi

Stems, leaves, and flowers were surface sterilized by immersing them in 0.1% Tween 20 for 30 s followed by treatment with 2% sodium hypochlorite for 5 min; they were washed in sterile water for 5 min. Subsequently, they were immersed in 70% ethanol for 5 min and then washed with sterile distilled water [12]. The stems, leaves, and flowers were cut into small pieces of 0.5 cm approximately and these sterilized tissue segments were inoculated on potato dextrose agar plates (PDA) (Difco™) supplemented with 50 μg/mL Kanamycin Sulfate (PanReac AppliChem, Darmstadt, Germany). The pieces of tissues of each plant were inoculated in triplicate in a Petri dish. A total of three plates were utilized by tissue. The plates were incubated for fourteen days in dark at 22 °C. Then, the fungi that emerged from the vegetal fragments were transferred to new plates with PDA medium and incubated at 22 °C. Then, each fungal species was subcultured several times to obtain a pure culture [12].

As surface sterilization control, 100 μL of water from the last wash was inoculated in a PDA plates. The plates were incubated at 22 °C. The absence of fungal growth on the culture media indicated that the sterilization of the plant segment surface was effective to eliminate the surface fungi [12]. The control was realized in triplicate.

The growth type of the obtained fungi was analyzed macroscopically by observing the colony on PDA medium and microscopically by observation of vegetative structures such as hyphae or yeasts by light microscopy (Optical Motic microscope, Hong Kong, China).

### 2.3. Antifungal Activity Determination against B. cinerea

In this study, the G29 isolate of *B. cinerea* was used. This isolate was originally obtained from a naturally infected grape (*Vitis vinifera*) [13].

#### 2.3.1. Dual Confrontation Assay

To evaluate the inhibitory effect on growth of *B. cinerea* of the endophytic fungi, dual confrontation assays were performed in PDA Petri dishes supplemented with 50 μg/mL Kanamycin Sulfate [14]. For the assays, mycelium discs (0.5 cm of diameter) of the endophytic fungus and *B. cinerea* were inoculated in a Petri dish. The mycelium discs of both fungi were placed 6 cm apart on culture medium in opposite sides of the plate. In the control, *B. cinerea* mycelium was inoculated in both sides of the plates [15]. After 10 days of incubation in the dark at 22 °C, the radial mycelial growth of *B. cinerea* towards the endophytic fungus (Ri) and radial mycelial growth of *B. cinerea* on a control plate (Rc) were measured and the percentage of inhibition was calculated according to the formula (Rc−Ri)/Rc × 100. The assays were performed four times with three replicates.

#### 2.3.2. Effect of Volatile Compounds on the Growth of *B. cinerea*

The effect of volatile compounds produced by the endophytic fungi on the growth of *B. cinerea* was examined using Sandwiched Petri plates assay; this culture technique prevents any direct contact between endophytic fungi and *B. cinerea* [16]. Endophyte fungi were inoculated in Petri plates containing PDA and were allowed to grow for 7 days at 22 °C. Then, *B. cinerea* was inoculated into another Petri plate containing PDA, and the base of Petri dish containing the endophytic fungus was put on top of the base of Petri dish containing *B. cinerea.* This system was completely sealed with parafilm to avoid the release of the volatile compounds. The effect on the volatile compounds on *B. cinerea* mycelial growth was evaluated by comparing the radial growth of the pathogen with the control corresponding to *B. cinerea* inoculated in the same conditions, but in the absence of the endophyte fungi. Inhibition percent was established as (R_1_ − R_2_/R_1_) × 100, where R_1_ is the distance covered by the pathogen in the control treatments, and R_2_ is the distance covered by the pathogen in the presence of the endophyte. All experiments were carried out in triplicate.

#### 2.3.3. Effect of Volatile Compounds on the Sporulation of *B. cinerea*

To determine if the volatile compounds secreted by endophytic fungi had an effect on sporulation of *B. cinerea*, the isolate Ec1 (isolated from *E. chiloensis*) was used. For this purpose, *B. cinerea* and the endophytic fungus were grown in Petri dishes containing PDA as it was described in Section 2.3.2. As negative control, only *B. cinerea* was used. After 7 days of incubation in the dark at 22 °C, *B. cinerea* conidia were removed from the Petri dishes by adding 3 mL of 0.9% NaCl and conidia were counted in a Neubahuer chamber. In this experiment, three independent experiments were performed, each one in triplicate.

#### 2.3.4. Effect of Diffusible Compounds on Growth and Germination of *B. cinerea*

Additionally, the effect of diffusible compounds produced by the isolate Ec1 on *B. cinerea* was analyzed by using the diffusion test assay or a cell-free filtrate.

For the diffusion test assay, the isolate Ec1 was inoculated in a Petri dish with PDA on a cellophane layer and incubated for 3 days at 22 °C. After this incubation time, cellophane containing the mycelium of the endophytic fungus was removed from the Petri dish and a 5-mm disc of *B. cinerea* mycelium was inoculated at the center of this Petri dish [17]. The culture was incubated for 5 days at 22 °C and radial growth was recorded and compared with that of the control. As control, a disc of *B. cinerea* mycelium from a 3-day old culture was inoculated in fresh culture medium. The inhibition of mycelial growth was evaluated by measuring the radial growth of *B. cinerea*. The percentage of inhibition was calculated as follows: % Inhibition = (R_1_ − R_2_) × 100/R_1_, where R_1_ is the growth distance of the *B. cinerea* in the control, and R_2_ corresponds to the distance of the growth of the pathogen in the diffusion test. The test was carried out in triplicate.

On the other hand, the effect of cell-free filtrates obtained from isolate Ec1 on germination of *B. cinerea* conidia was evaluated. To obtain cell-free filtrates, the isolate Ec1 was inoculated into a 250 mL flask containing 100 mL of PDB (Potato Dextrose Broth). The culture was incubated at 22 °C with agitation (140 rpm) for 12 days. The culture was centrifuged at 5200× *g* for 30 min at 4 °C to collect the supernatant and, the supernatant was filtered with a 0.22-µm filter [18]. To determine the effect of the cell-free filtrate on conidial germination, the assay was executed as described [19]. A conidial suspension of *B. cinerea* at a concentration of 1 × 10^3^ conidia mL^−1^ was inoculated in 24-well plates containing PDB medium and the cell-free filtrate at 50 or 100% supplemented with 1% glucose. As germination control, the suspension of conidia of *B. cinerea* was inoculated in PDB medium. The cultures were incubated for 4 h at 22 °C. After this time of incubation, percentage of germination was determined as cg/ct × 100, where cg are the germinated conidia and ct are the total conidia. The conidia were considered germinated when germ tube length was equal or greater than conidial diameter. The rate of germ tube growth (measured in µm/h) after 4 h of incubation was also determined. In both experiments at least 100 conidia were photographed for each concentration of cell-free filtrate and for the control. The images were captured with a digital camera Moticam 2300 (Hong Kong, China) coupled to a light Motic Microscope and the images were analyzed with Motic Image Plus 2.0 software. These experiments were carried out in triplicate.

To detect the diffusible compounds with antifungal activity produced by the isolate Ec1, thin-layer chromatography (TLC) coupled with bioautographic was performed [20]. For this purpose, the isolate Ec1 was inoculated in PDB medium and was incubated for 12 days at 22 °C with agitation (140 rpm). Then, the culture was centrifuged at 5200× *g* for 30 min at 4 °C to separate the fungal biomass, the pellet was discarded and the supernatant was filtered with a 0.22-µm filter. Then, filtrated supernatant was subjected to liquid–liquid partition with ethyl acetate three times [20]. As control, an ethyl acetate extraction was carried out from PDB culture medium without inoculation with the fungus. The extracts were dried using a rotary evaporator.

Subsequently, compounds of the extracts were separated on aluminum-backed TLC plates (silica gel 60 F_254_, Merck, Santiago, Chile). The TLC plates were developed under saturated conditions with the system chloroform/methanol (95:5) as eluent. Compounds on the plates were visualized under UV light at 254 nm. To detect the compounds with antifungal activity on the TLC plate, a bioautography was conducted [20]. For this, the developed plates were dried to eliminate the solvent. The dry plate was placed in a Petri dish on PDA medium supplement with 50 µg/mL Kanamycin Sulfate. Then, 3 mL of a conidial suspension of *B. cinerea* (1 × 10^6^ conidia/mL) was poured on the plate. Cultures were incubated for 7 days at 22 °C.

### 2.4. Identification of Endophytic Fungi

For the identification of the fungi, morphological and molecular techniques were used. For morphological identification, microcultures were performed. Sterile pieces 2 cm^2^ of PDA were placed in a slide and were inoculated with the isolated endophytic fungi and then the PDA pieces were covered with a coverslip. Then, the slides containing the inoculated solid culture medium were put inside sterile Petri dishes containing filter paper with glycerol 30% to maintain humidity. The fungal cultures were incubated for 7 to 10 days at 22 °C until the formation of the aerial structures. The visualization of the structures was performed using a light Motic microscope Hong Kong, China.

For the identification at genus level of the endophyte fungi, comparison of the ITS regions of the rDNA was used. For this, mycelia obtained from axenic cultures were inoculated on a cellophane paper layer placed on Petri dishes with PDA. The culture was incubated for 3 days at 22 °C. Then, the fungal mycelium was recovered and the genomic DNA was obtained using the CTAB method [21] with some modifications. For this, approximately 200 mg of fungal tissue was deposited in a falcon tube, then 800 µL of CTAB buffer (3% CTAB, 100 mM Tris-HCl pH 8.0, 1.4 M NaCl, 20 mM EDTA) was added to the tube and then the tissue was ground with glass balls of 4 mm diameter and vortex for 3 min. The ground tissue was incubated at 60 °C for 30 min. Then, the cellular debris was centrifuged at 5000× *g* for 10 min at room temperature and the supernatant was recovered. Subsequently, an equal volume of chloroform: isoamyl alcohol (24: 1) was added. The mixture was centrifuged at 7000× *g* for 10 min at 4 °C and the aqueous phase was recovered, an equal volume of cold isopropanol was added and, the solution was incubated at −20 °C for 2 h. Then, 500 µL of absolute ethanol was added to the pellet and incubated for 2 h at −20 °C, the suspension was centrifuged at 7000× *g* for 10 min at 4 °C and the supernatant was discarded. Finally, 50 µL of DNAse free H_2_O was added and the DNA was stored at −20 °C until use.

The ITS sequences were amplified using the ITS-1 and ITS-4 primers forward primer was ITS-1 5′-TCCGTAGGTGAACCTGCGG-3′and reverse primer 5′-TCCTCCGCTTATTGATATGC-3′ [22]. The PCR reaction was performed in a volume of 50 μL containing; 2 μL of genomic DNA, 1 μL of ITS-1 primer (10 μM), 1 μL of ITS-4 primer (10 μM), 25 μL of GoTaq^®^ Green Master Mix 2x (Promega, Madison WI, USA) and 21 μL of nuclease-free H_2_O. The PCR program consisted of an initial denaturation at 94 °C for 3 min, then 38 cycles consisting of 94 °C for 40 s, followed by 57 °C for 40 s and 72 °C for 40 s, finally an elongation at 72 °C for 5 min. The PCR products were sequenced by Macrogen Inc. (Seoul, Korea) in both directions

Fungal ITS region sequences from endophytes were manually edited with Geneious Prime^®^ 2020.0.4 software. Final nucleotide sequence of the isolates Lc1, Ac1 and Ec1 were submitted to GenBank NCBI using the Basic Local Alignment Search Tool (BLAST) [23]. Sequences were aligned against fungi ITS sequences from NCBI, and BLASTn tool was used to search sequences that presented similarity with them. Consensus sequences tree construction was achieved with Geneious Prime^®^ 2020.0.4 software, and the closest species sequences obtained from GenBank were selected to perform the alignment. The sequences used are detailed in Table S1. Clustal Ω algorithms were used, and dendograms were constructed using Tamura-Nei genetic distance model with neighbor-joining method. The resampling for each dendrogram was 1000 bootstrap and the support threshold was of 50% [24].

### 2.5. Statistical Analysis

All the statistical analyses were conducted using GraphPad Prism 5.0. To determine the effects of the different treatments, Analysis of variance (ANOVA) with Tukey’s test was carried out. Means were separated with the least significant difference test (*p* < 0.05).

## 3. Results

### 3.1. Isolation of Endophytic Fungi

From all of endemic and native plants studied, it was possible to isolate endophytic fungus. A total of eleven endophyte isolates were obtained (Table 1). Among these, eight corresponded to filamentous fungi and three to yeast-like fungi. In general, one endophyte was isolated from most of the plants, with exception of *T. tricolor*.

To evaluate the antifungal activity against *B. cinerea* of isolated endophytic fungi, a confrontation assay was used. From these fungi, only the isolates Lc1, Ac1 and Ec1 inhibited the mycelial growth of *B. cinerea* (Table 2). These isolates exhibited a similar antifungal effect on the mycelial growth of *B. cinerea.*

**Figure 1:**
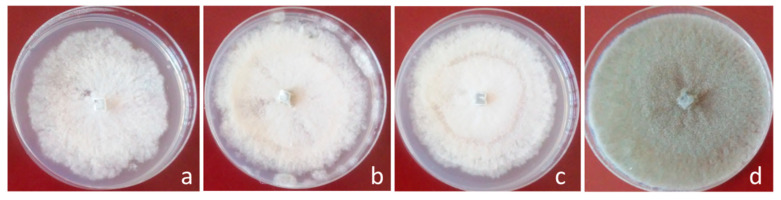
Macroscopic morphology of *B. cinerea* mycelia after 7 days of incubation in the presence of volatile compounds produced by the isolates Lc1 (**a**), Ac1 (**b**) and Ec1 (**c**). *B. cinerea* growth in the absence of volatile compounds (**d**).

In addition, the antifungal effect against *B. cinerea* of volatile compounds produced by the endophytic fungi was evaluated (Table 2). The three fungi inhibited the mycelial growth of *B. cinerea* through the production of volatile compounds. The antifungal effect against *B. cinerea* produced by the volatile compounds from the three endophytic fungi was similar. In addition, phenotypical differences in the *B. cinerea* mycelium were observed when the fungus was exposed to volatile compounds produced by the endophytic fungi (Figure 1). The *B. cinerea* mycelium without treatment with volatile compounds showed a gray coloration due to the presence of conidia, on the other hand, when *B. cinerea* was exposed to the volatile compounds produced by the isolates Lc1, Ec1 or, Ec1, the mycelia remained white, suggesting that the volatile compounds would affect the sporulation of *B. cinerea.*

**Figure 2:**
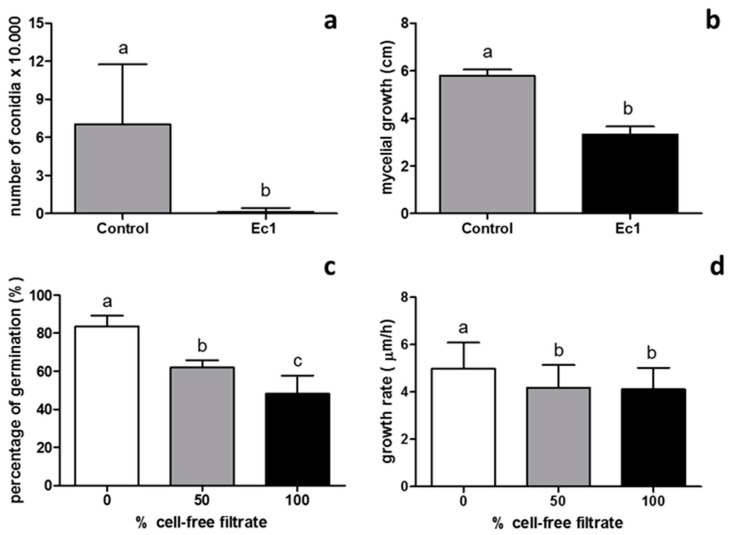
Antifungal activity of volatile and diffusible compounds secreted by the isolate Ec1 against *B. cinerea*. (**a**) Effect of volatile compounds secreted by Ec1 on sporulation of *B. cinerea* after 7 days of incubation at 22 °C. (**b**) Effect of diffusible compounds on mycelial growth of *B. cinerea* after 3 days of incubation at 22 °C. Gray bars represent *B. cinerea* control and black bars represent isolate Ec1 test treatment. (**c**) Effect of the cell-free filtrate obtained from Ec1 at different concentrations on germination of *B. cinerea* conidia after 4-h incubation. (**d**) Growth rate (µm/h) of the germ tube of *B. cinerea* after 4 h of incubation at different concentration of cell-free filtrates obtained from Ec1. Values represent the mean ± SD. a, b: A Student’s *t*-test and Mann Whitney test were completed. c, d: Analysis of variance (ANOVA) with Tukey’s test was carried out. Columns with different letters are significantly different (*p* < 0.05).

To quantify the effect of volatile compounds on *B. cinerea* sporulation, the fungus was cultivated in the presence or absence of volatile compounds produced by the isolate Ec1. Then, conidia were extracted from the culture media of *B. cinerea* and were quantified. The volatile compounds produced by isolate Ec1 reduced the sporulation of *B. cinerea* by 98.4% (Figure 2a).

To determine if the effect of the endophytic fungi on the mycelial growth of *B. cinerea* in the confrontation assays was due to the secretion of diffusible compounds, diffusion test assays and a study using a cell-free filtrate were realized.

For the diffusion test assay, the isolate Ec1 was inoculated in a Petri dish with PDA on a cellophane layer and incubated for 3 days. After this incubation time, cellophane containing the mycelium of the endophytic fungus was discarded and the effect on *B. cinerea* of the compounds secreted by the endophytic fungus to the culture medium was determined (Figure 2b). The compounds secreted by the isolate Ec1 to the culture medium inhibited the mycelial growth in about 42.1%. Correspondingly, an antifungal assay using a cell-free filtrate obtained from liquid medium culture of the isolate Ec1 was performed. This filtrate was used to evaluate their effect on conidial germination of *B. cinerea* (Figure 2c). The results show that the cell-free filtrate obtained from isolate Ec1 at 50 or 100% inhibited the germination in 25.8% and 42.3%, respectively. Moreover, the effect of the cell-free filtrate on the elongation of the germ tube was evaluated (Figure 2d). A decrease in the germ tube growth with respect to control was observed. The growth rate of germ tube in the absence cell-free filtrate was 5 µm/h, and in the presence of cell-free filtrate at 50 or 100% was 4.19 µm/h or 4.11 µm/h, respectively. Therefore, the isolate Ec1 was able to secrete diffusible compounds affecting the mycelia growth, conidia germination and elongation of germ tube.

**Figure 3:**
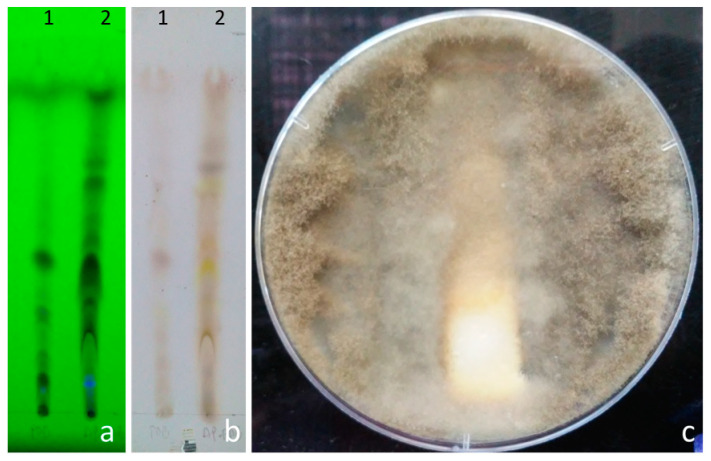
Compounds extracted from Ec1 isolate and their antifungal activity on *B. cinerea*. (**a**) Chromatogram of the extract visualized under UV light at 254 nm. (**b**) Chromatogram of the extract revealed with 25% H_2_SO_4_. Lane 1, compounds obtained from the culture medium in the absence of isolate Ec1. Lane 2, compounds secreted by the isolate Ec1. Mobile phase CHCl_3_/MeOH 95: 5. (**c**) Bioautography of the extract obtained from Ec1 after 7 days of *B. cinerea* incubation at 22 °C.

Figure 3a,b show chromatograms of the compounds secreted by the isolate Ec1. Additionally, chromatograms of the compounds extracted from the culture medium without inoculating the fungus are shown. The isolate Ec1 secreted several compounds which were different to those extracted from culture medium without inoculation with this fungus. It was also determined, through a bioautography analysis, that only some compounds secreted by the isolate Ec1 had antifungal activity against *B. cinerea.* An inhibition halo on the chromatogram is observed in the zone corresponding to migration of the more polar compounds (Figure 3c).

### 3.2. Morphological Identification of Endophytic Fungi with Antifungal Activity

**Figure 4:**
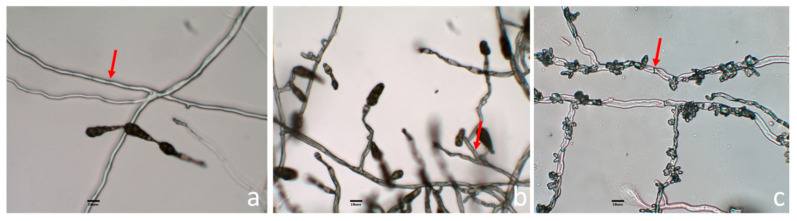
Morphological examination of microscopic aerial structures from Ac1 (**a**), Lc1 (**b**) and, Ec1 (**c**). The aerial structures of the three fungi were observed with the 40× objective. The septa are indicated by a red arrow.

For the identification of the fungi, morphological and molecular techniques were used. According to the microscopic observation, the isolate Ac1 presented branched septate hyphae and the conidia were ovoid or ellipsoidal with horizontal and vertical septa and were produced in chains (Figure 4a). The isolate Lc1 also presented septate hyphae and the conidia were similar to those observed in Ac1, but concatenated conidia were not observed (Figure 4b). The Ec1 isolate showed septate hyphae. The hyphae contained conidia in clusters which were located along the hyphae (Figure 4c).

**Figure 5:**
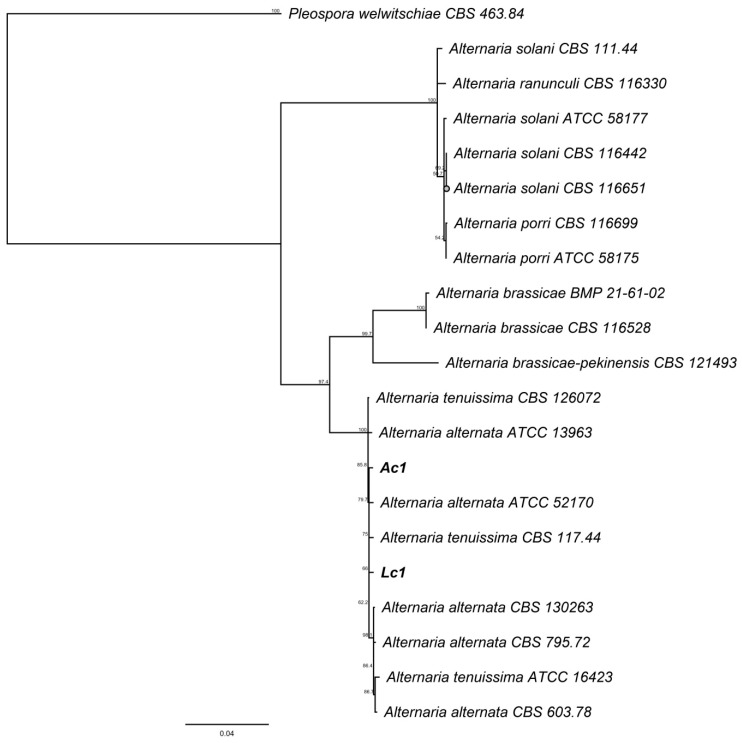
Neighbor-joining tree based on fungal internal transcribed spacer (ITS) sequences of Ac1, Lc1, 15 *Alternaria* spp. and *Pleospora welwitschiae* as outgroup. Numbers labeled at each node indicate bootstrap value (%) from 1000 replicated.

**Figure 6:**
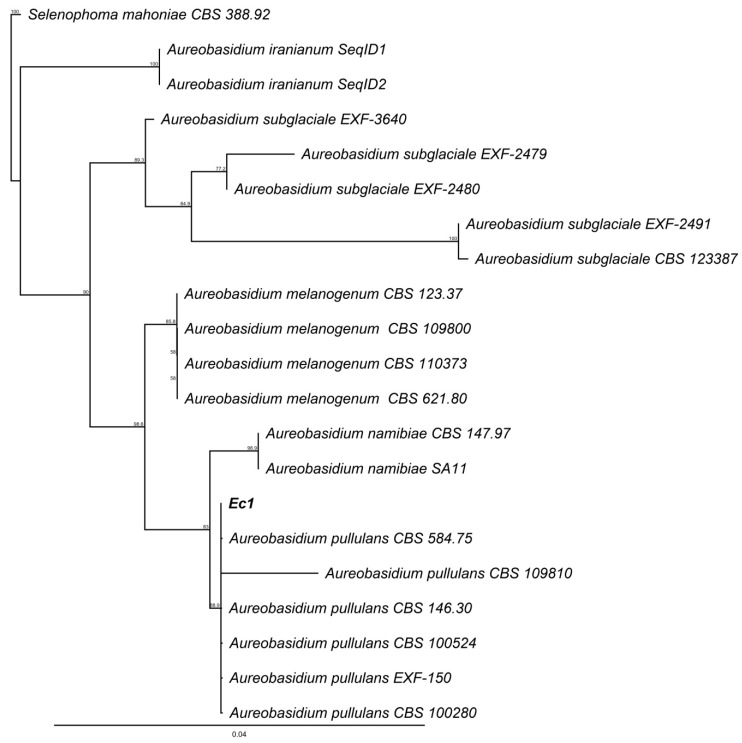
Neighbor-joining tree based on fungal internal transcribed spacer (ITS) sequences of Ec1, *Aureobasidium* spp. and *Selenophoma mahoniae* as outgroup. Numbers labeled at each node indicate bootstrap value (%) from 1000 replicated.

Morphological analysis of the isolates was complemented by phylogenetic analysis of ITS markers, the Neighbor-joining tree generated from the data is presented in the Figure 5 for Lc1 and Ac1 sequences and, in the Figure 6 for Ec1 sequence. The trees generated were made by Neighbor-joining using BLAST alignment with NCBI database.

The isolates Lc1 and Ac1 showed conidia morphology similar to *Alternaria* genus [25,26]. Neighbor-joining tree of these fungi were clustered with *Alternaria* species which includes *A. alternata* and *A. tenuissima* [27,28]. Therefore, the conidia morphology and the phylogenetic analysis support the assignation of these fungi to this genus.

On the other hand, phylogenetic analyses based on internal transcribed spacer (ITS) allowed the identification of the isolate Ec1 as *Aureobasidium* spp. (Figure 6). This isolate showed morphology similar to yeast-like fungus *A. pullulans*; this fungus changes between its filamentous and yeast form [29].

## 4. Discussion

In this work, three endophytic fungi (Ac1, Lc1 and Ec1) with antifungal activity against *B. cinerea* obtained from native and endemic plants growing in Central Andean Precordillera of Chile were isolated. This region has a Mediterranean climate [9,10,30]. It has been described that the plant colonization by endophytic fungus is dependent on the ecosystem. Colonization rate was from less than 1 to 44% in arctic and boreal ecosystems to more than 90% in tropical ecosystems [31]. Endophyte communities along a latitudinal gradient from the Canadian arctic to the tropical forest of central Panama were studied and it was shown that endophytic fungi increase in incidence from arctic to tropical sites [32]. These authors defined the leaves of tropical trees as hotspots for fungal species diversity [32]. Little is known about the abundance of endophytic fungi in Mediterranean ecosystems. Some studies have been conducted in oaks and pines [33,34,35,36]. Additionally, a total of 319 fungal species were obtained from roots of 24 plant species from the Province of Alicante (Spain). *Fusarium* and *Phoma* species were the most abundant genera, followed by *Aspergillus*, *Alternaria* and *Acremonium* [37]. These results coincide with those found in this work since *Alternaria* was found as endophytic fungus from two different plants. However, in this work low numbers of the isolates of endophytic fungi were obtained. The rigorous conditions of disinfection of the plant surface to eliminate the epiphytic fungi could explain this result.

This is the first report of endophytic fungi isolated from plants located in the studied area and it is also the first time that endophytic fungi with antifungal activity against *B. cinerea* have been isolated from *L. caustica, A. caven and E. chiloensis*. *L. caustica* (litre) is an endemic tree of Chile and *E. chiloensis* is an endemic columnar cactus species widely distributed in north-central Chile that inhabits mainly equatorial-facing slopes in semiarid environments [30,38]. *A. caven* is a native tree found in several countries in South America [39]. Although endemic or native plants of this region were used in this work, the isolated fungi *Alternaria* spp. (Lc1 from *L. caustica*), *Alternaria* spp. (Ac1 from *A. caven*) and *Aureobasidium* spp. (Ec1 from *E. chiloensis*) have been commonly found as endophytes in different types of plants around the world [12,40]. *Alternaria* and *Aureobasidium* genera have been isolated as endophytes from different cacti species in Arizona [12] and from grapevine cultivars in Canary Islands [40].

In Chile, some studies have reported the presence of endophytic fungi in native, endemic and introduced plants [41,42,43,44]. The endophytic fungi *Microsphaeropsis olivacea* and *Penicillium janczewskii* isolated from native gymnosperms collected in southern Chile showed antifungal activity on *B. cinerea* [43].

Species from genus *Alternaria* have been described as saprobic, endophytic, and pathogenic [45]. Many species of *Alternaria* isolated as endophytic produce antifungal compounds [39]. Some of these species have been reported with activity against *B. cinerea* [40,46,47]. On the other hand, it has been reported that *A. pullulans* is an important antagonist of *B. cinerea* [15,48,49]. *Alternaria* and *Aureobasidium* species are able to inhibit the mycelial growth of *B. cinerea* by antibiosis mediated by diffusible compounds [50,51,52] and, in the case of *A. pullulans* also through the production of volatile compounds [15,49,53]. Indeed, *A. pullulans* is the active ingredient of fungicides that are currently commercialized against *B. cinerea* [7].

In this work, the results suggest that the isolated endophytic fungi would inhibit to *B. cinerea* through the secretion of diffusible and volatile compounds affecting the mycelial growth. In the case of the isolate Ec1 corresponding to *Aureobasidium* spp., it was also demonstrated that extracts obtained from this fungus inhibited the mycelial growth, the conidia germination and the elongation of the germ tube. The compounds secreted by *Aureobasidium* spp. were not identified, but it was shown that the most polar compounds of this extract exhibited the antifungal activity.

Interestingly, in this work it was also shown that the volatile compounds produced by the three isolated endophytic fungi suppressed the sporulation of *B. cinerea*. There are no works published in the literature describing the effect of volatiles produced by *Alternaria* or *Aureobasidium* species on the sporulation of *B. cinerea*. Reduction of sporulation is a crucial step for controlling *B. cinerea* disease cycle in plants.

*A. pullulans* presents great phenotypical variability. In some varieties nine secondary metabolite biosynthetic clusters were identified. However, in other varieties 32 or 37 biosynthetic clusters have been identified [54] therefore, it is possible that the isolate Ec1 might produce new compounds with antifungal activity that have not been previously described.

## 5. Conclusions

Three endophyte fungi with antifungal activity against *B. cinerea* were isolated form endemic and native plants of Chile. The antifungal activity is mediated by diffusible and volatile compounds which reduced mycelial growth, sporulation and conidia germination of *B. cinerea*.

## Figures and Tables

**Table 1:** Endophytic fungi isolated from plants growing in Central Andean Precordillera of Chile.

Plant	Isolation Organ	Fungal Isolate	Growth Type
*C. polifolia*	Leaf	Cp1	Filamentous
*L. caustica*	Leaf	Lc1	Filamentous
*C. odorifera*	Leaf	Co1	Filamentous
*E. chiloensis*	Stem	Ec1	Filamentous
*T. tricolor*	Flower	Tt1	Filamentous
	Stem	Tt2	Filamentous
	Flower	Tt3	Yeast
	Stem	Tt4	Yeast
*P. chilensis*	Flower	Pc1	Filamentous
	Stem	Pc2	Yeast
*A. caven*	Stem	Ac1	Filamentous

**Table 2:** Effect of endophytic fungus on *B. cinerea* mycelial growth in confrontation assays and by volatile compounds.

Fungal Endophyte	Inhibition of Mycelial Growth in the Confrontation Test (%)	Inhibition of Mycelial Growth Produced by Volatile Compounds (%)
Lc1	36.0 ± 4.0	22.4 ± 3.5
Ac1	41.9 ± 2.8	18.0 ± 3.8
Ec1	36.6 ± 4.5	17.0 ± 1.5

Mean of four independent experiments ± the standard deviation.

## Supplementary Materials

The following are available online at https://www.mdpi.com/2309-608X/6/3/149/s1.
