# Supplementary material for: Endophytic Fungi Isolated from Plants Growing in Central Andean Precordillera of Chile with Antifungal Activity against Botrytis cinerea
Source: J Fungi (Basel). 2020 Aug 26;6(3):149. doi: 10.3390/jof6030149 (PMC7560031; doi:10.3390/jof6030149)
Supplement: Supplementary file 1 [file jof-06-00149-s001.pdf]

Table S1. Sequences used for identification analyses of the endophytic fungi

Organism	Strain	GenBank accession no.	Reference
Pleospora welwitschiae	CBS 463.84	MH861766.1	*Stud. Mycol.* 92, 135-154 (2019)
Selenophoma mahoniae	CBS 388.92	FJ150872.1	*Stud. Mycol.* 61, 21-38 (2008)
Alternaria spp.			
A. solani	ATCC 58177	AF229475.1	*Mycol. Res.* 104 (11), 1312-1321 (2000)
A. ranunculi	CBS 116330	KJ718225.1	*Stud. Mycol.* 79, 1-47 (2014)
A. solani	CBS 116442	KJ718240.1	*Stud. Mycol*. 79, 1-47 (2014)
A. solani	CBS 116651	KC584217.1	*Stud. Mycol*. 75 (1), 171-212 (2013)
A. porri	ATCC 58175	AF229470.1	*Mycol. Res*. 104 (11), 1312-1321 (2000)
A. porri	CBS 116699	KJ718218.1	*Stud. Mycol*. 79, 1-47 (2014)
A. solani	CBS 111.44	Y17070.1	Unpublished
A. brassicae	BMP 21-61-02	AF229463.1	*Mycol. Res*. 104 (11), 1312-1321 (2000)
A. brassicae-pekinensis	CBS 121493	KC584244.1	*Stud. Mycol.* 75 (1), 171-212 (2013)
A. brassicae	CBS 116528	KC584185.1	*Stud. Mycol.* 75 (1), 171-212 (2013)
A. tenuissima	CBS 126072	MH864067.1	*Stud. Mycol.* 92, 135-154 (2019)
A. alternata	ATCC 52170	FJ545250.1	Unpublished
A. tenuissima	CBS 117.44	MH856117.1	*Stud. Mycol*. 92, 135-154 (2019)
A. alternata	CBS 130263	KP124390.1	*Stud. Mycol.* 82, 1-21 (2015)
A. alternata	CBS 795.72	KP124309.1	*Stud. Mycol*. 82, 1-21 (2015)
A. tenuissima	ATCC 16423	AF229476.1	*Mycol. Res.* 104 (11), 1312-1321 (2000)
A. alternata	CBS 603.78	KP124312.1	*Stud. Mycol.* 82, 1-21 (2015)
Aureobasidium spp.			
A. iranianum	clone SeqID1	MG722806.1	*New Dis. Rep.* 37, 4 (2018)
A. iranianum	clone SeqID2	MG722807.1	*New Dis. Rep.* 37, 4 (2018)
A. subglaciale	EXF-3640	FJ150896.1	*Stud. Mycol*. 61, 21-38 (2008)
A. subglaciale	EXF-2479	FJ150893.1	*Stud. Mycol*. 61, 21-38 (2008)
A. subglaciale	EXF-2491	FJ150894.1	*Stud. Mycol*. 61, 21-38 (2008)
A. subglaciale	CBS 123387	NR_147323.1	*Stud. Mycol*. 61, 21-38 (2008)
A. melanogenum	CBS 123.37	MH855849.1	*Stud. Mycol*. 92, 135-154 (2019)
A. melanogenum	CBS 109800	FJ150880.1	*Stud. Mycol.* 61, 21-38 (2008)
A. melanogenum	CBS 110373	FJ150887.1	*Stud. Mycol*. 61, 21-38 (2008)

A. melanogenum	CBS 621.80	FJ150885.1	*Stud. Mycol*. 61, 21-38 (2008)
A. namibiae	CBS 147.97	AJ244231.1	Unpublished
A. namibiae	SA11	MF398842.1	Unpublished
A. pullulans	CBS 584.75	FJ150906.1	*Stud. Mycol*. 61, 21-38 (2008)
A. pullulans	CBS 109810	FJ150901.1	*Stud. Mycol*. 61, 21-38 (2008)
A. pullulans	CBS 146.30	FJ150902.1	*Stud. Mycol*. 61, 21-38 (2008)
A. pullulans	CBS 100524	FJ150905.1	*Stud. Mycol*. 61, 21-38 (2008)
A. pullulans	strain EXF-150	FJ150908.1	*Stud. Mycol.* 61, 21-38 (2008)
A. pullulans	CBS 100280	FJ150910.1	*Stud. Mycol*. 61, 21-38 (2008)
